# Classifying Residual Stroke Severity Using Robotics-Assisted Stroke Rehabilitation: Machine Learning Approach

**DOI:** 10.2196/56980

**Published:** 2024-10-07

**Authors:** Russell Jeter, Raymond Greenfield, Stephen N Housley, Igor Belykh

**Affiliations:** 1 Department of Mathematics and Statistics Georgia State University Atlanta, GA United States; 2 Motus Nova, LLC Atlanta, GA United States; 3 Laboratory for Sensorimotor Integration Georgia Institute of Technology Atlanta, GA United States; 4 Neuroscience Institute Georgia State University Atlanta, GA United States

**Keywords:** stroke, rehabilitation robotics, machine learning, artificial intelligence, physical therapy, neuroplasticity

## Abstract

**Background:**

Stroke therapy is essential to reduce impairments and improve motor movements by engaging autogenous neuroplasticity. Traditionally, stroke rehabilitation occurs in inpatient and outpatient rehabilitation facilities. However, recent literature increasingly explores moving the recovery process into the home and integrating technology-based interventions. This study advances this goal by promoting in-home, autonomous recovery for patients who experienced a stroke through robotics-assisted rehabilitation and classifying stroke residual severity using machine learning methods.

**Objective:**

Our main objective is to use kinematics data collected during in-home, self-guided therapy sessions to develop supervised machine learning methods, to address a clinician’s autonomous classification of stroke residual severity–labeled data toward improving in-home, robotics-assisted stroke rehabilitation.

**Methods:**

In total, 33 patients who experienced a stroke participated in in-home therapy sessions using Motus Nova robotics rehabilitation technology to capture upper and lower body motion. During each therapy session, the Motus Hand and Motus Foot devices collected movement data, assistance data, and activity-specific data. We then synthesized, processed, and summarized these data. Next, the therapy session data were paired with clinician-informed, discrete stroke residual severity labels: “no range of motion (ROM),” “low ROM,” and “high ROM.” Afterward, an 80%:20% split was performed to divide the dataset into a training set and a holdout test set. We used 4 machine learning algorithms to classify stroke residual severity: light gradient boosting (LGB), extra trees classifier, deep feed-forward neural network, and classical logistic regression. We selected models based on 10-fold cross-validation and measured their performance on a holdout test dataset using *F*_1_-score to identify which model maximizes stroke residual severity classification accuracy.

**Results:**

We demonstrated that the LGB method provides the most reliable autonomous detection of stroke severity. The trained model is a consensus model that consists of 139 decision trees with up to 115 leaves each. This LGB model boasts a 96.70% *F*_1_-score compared to logistic regression (55.82%), extra trees classifier (94.81%), and deep feed-forward neural network (70.11%).

**Conclusions:**

We showed how objectively measured rehabilitation training paired with machine learning methods can be used to identify the residual stroke severity class, with efforts to enhance in-home self-guided, individualized stroke rehabilitation. The model we trained relies only on session summary statistics, meaning it can potentially be integrated into similar settings for real-time classification, such as outpatient rehabilitation facilities.

## Introduction

Stroke is a leading cause of mortality and disability worldwide, and the economic costs of treatment and poststroke care are substantial [1]. In 2019, there were 12.2 million incident cases of stroke, 101 million prevalent stroke cases, and 6.55 million deaths from stroke [2]. The severity of a stroke can range from mild to severe, with severe strokes often leading to long-term disability or even death. Stroke rehabilitation typically involves a team of health care professionals, including doctors, nurses, therapists, and other specialists. The specific goals and interventions of stroke rehabilitation vary depending on the individual’s needs and abilities. They may include physical therapy to improve mobility; occupational therapy to improve the ability to perform daily activities; speech therapy to improve communication skills; and cognitive therapy to improve memory, problem-solving, and other cognitive abilities. While traditionally recovery has taken place in inpatient and outpatient rehabilitation facilities, there is growing recent literature about moving the recovery process into the home [3,4] and integrating technology-based interventions [5]. This study takes steps to achieve this goal of in-home and autonomous recovery for patients who experienced a stroke via robotics-assisted stroke rehabilitation and classification of stroke residual severity via machine learning methods.

Machine learning in health care and stroke rehabilitation is not a new concept (see Reyna et al [6], Alabi et al [7], Cerasa et al [8], and Harari et al [9] as notable examples of this vast research field and Campagnini et al [10] for a systematic review of machine learning methods for poststroke rehabilitation recovery prediction). In particular, multiple studies have been performed to predict outcomes in patient survival, locoregional recurrences, and long-term outcomes in patients who experienced an ischemic stroke [11-15]. Similarly, studies focused on motor function have leveraged retrospective health care data and targeted predicting the short- and long-term functional ability [16-18]. Such studies represent an exciting step forward in stroke rehabilitation but have some limitations. These limitations include the use of health care data that are infrequently measured (sometimes entirely limited to admission data), which can hamper the performance of models that rely on large datasets for generalizability. Similarly, most studies limit their scope to predicting short- and long-term outcomes and may fail to capture some of the day-to-day changes survivors’ who have experienced a stroke experience.

This study aims to overcome these limitations by quantifying the progress of patient improvement via in-home therapy sessions using Motus Nova robotics rehabilitation technology [19] that captures upper and lower body motion. The Motus Hand and Motus Foot devices are robotic therapeutic devices designed to be used by survivors who have experienced a stroke with residual upper and lower extremity impairments at home without needing help from a clinician or caregiver. The neuromotor mechanism by which the Motus Hand and Motus Foot help rehabilitate patients who have experienced a stroke is rooted in the results from constraint-induced movement therapy studies [20,21] and focus on getting survivors of stroke high volumes of repetitive task practice. The Motus Hand and Motus Foot engage the affected wrist or ankle of the user, guiding them through various therapeutic exercises targeting various functional tasks (eg, gross motor control, fine motor control, and precision tracking). Earlier versions of the technology have been shown to have clinically significant improvements in depressive symptoms, functional independence, upper extremity use in functional tasks, distance walking, and gait speed [19,22,23].

Traditionally, to determine the functional ability of survivors of stroke, they will be assessed by a clinician during often infrequent clinical visits (whether through an outpatient rehabilitation facility, visiting a neurologist, or a primary care physician). The time scale of these assessments fails to capture the progress made during the recovery process when it happens. Using machine learning and therapy session, kinematic measurements promise to have a central role in rehabilitation decision-making in determining whether patient therapy is improving. Machine learning is the methodology that allows computers to learn from experience. By constructing and training supervised classifiers to learn decision rules from data, automatic solutions can be exploited to make predictions on new data [24,25]. As in many health care, disease, or machine learning research applied in a clinical setting, labeling of patient data by a clinician is necessary [6]. This study applies the same heuristic methodologies. Our goal is to use kinematics data collected during in-home, self-guided therapy sessions to construct supervised machine learning methods to address the autonomous classification of stroke residual severity–labeled data toward improving in-home, robotics-assisted, individualized stroke rehabilitation.

## Methods

### Therapeutic Intervention Description

**Figure 1:**
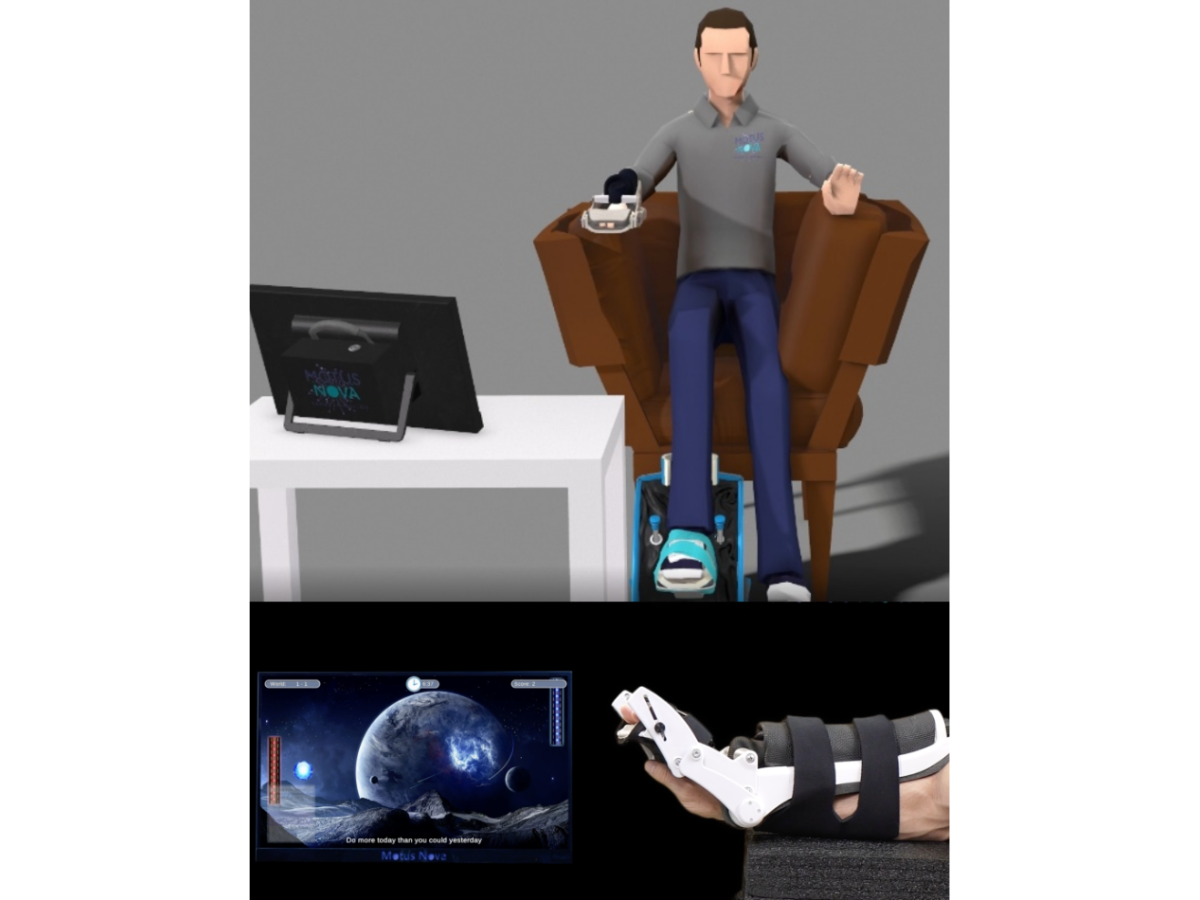
Patients do therapy sessions with the Motus Hand or Motus Foot using a pneumatically driven exogenous robotic device worn on the affected hand, arm, or foot (the Motus Hand is depicted in the bottom panel). The peripheral acts as a game controller (through an angle sensor embedded in the wrist joint) that allows users to play therapeutic video games that dynamically adapt to their needs and provide the requisite assistance or resistance (computer screen in the bottom panel).

The Motus Hand and Motus Foot each consist of 2 major components: a peripheral (see the bottom panel of Figure 1 for a close-up of the Motus Hand peripheral) that the patient attaches to their affected limb and a console that guides their therapy routine and assessment using a video game interface. The peripherals have a pneumatic actuator that can dynamically provide assistance or resistance by filling an air muscle in the peripheral that moves the wrist or ankle joint. The wrist or ankle joint of the peripheral has an embedded angle and pressure sensor that transmits live angle and pressure data to the console. This allows the console to give the user immediate visual feedback of their movement through avatars in a video game on the screen. The therapeutic video game activities can provide a dynamic feedback loop consisting of in-game goals (eg, ships to shoot or coins to collect) that drive user movements, which correspond to movement on the screen, allowing the console to react and set new goals or obstacles. This feedback loop is designed to promote sensory motor function.

A therapy session with the Motus Hand or Motus Foot consists of stretching, gross motor control, fine motor control, and endurance exercises, depending on the patient’s needs. This process is depicted in Figure 1, where a Motus Hand user is playing “Cosmic Tennis,” a gross motor control exercise that plays like the classic arcade game Pong [26]. The user’s wrist or ankle movement corresponds to the movement of the paddle on the right-hand side of the screen, and the goal is to hit the ball back and forth to score on the artificial intelligence (AI)–controlled opponent. Because of the user-guided nature of a therapy session with the Motus Hand or Motus Foot, therapy sessions can vary greatly in length. In the data collected, therapy sessions range from 5 to 60 minutes and between 1 and 10 therapeutic activities.

### Study Design

The Motus Hand and Motus Foot collect high-resolution angle and pressure data from sensors embedded in the wrist or ankle joints and the pressure management system. These high-resolution data are collected at a frequency of 30 Hz and stored in a time series database. Other information collected during a therapy session includes score, peripheral type (Motus Hand or Motus Foot), and current game (therapeutic activity). This study used anonymous data collected from 33 patients who experienced a stroke. In total, those patients performed 32,902 therapeutic activities (ie, each unique activity performed in each therapy session). These therapy session data are then divided using an 80%:20% split into a training dataset and a holdout test set. The training set is used for training the classification models, and the test set is reserved for the final model evaluation.

To use the data collected during a therapy session to classify a patient’s stroke residual severity autonomously, each patient was given a guided assessment with a clinician using the Motus Hand or Motus Foot to classify them as having a high range of motion (ROM), low ROM, or no ROM. These classification levels are intentionally chosen to be coarse to mimic the environment in a rehabilitation therapy session.

**Figure 2:**
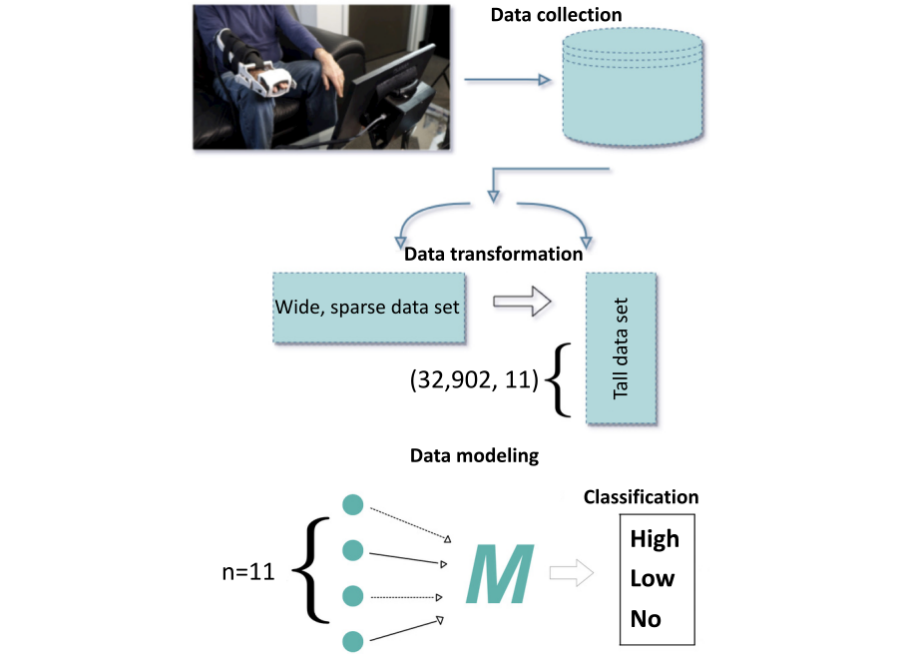
Concept diagram of the overall data analysis and modeling. In total, 33 patients perform in-home therapy using the Motus Hand and Motus Foot rehabilitation devices. Sensors in the devices capture live angle and pressure data. These data are then processed and summarized to provide summary statistics of 32,902 therapeutic activities. This provides the base dataset for the analysis presented in this paper, with 11 features and 32,902 points. These data are then split and prepared for use in training a supervised machine learning model to classify the stroke severity of the patient.

To find an ideal classifier, we use to consider the training and performance of 4 machine learning algorithms: light gradient boosting (LGB) [27], extra trees classifier [28], deep feed-forward neural network (DNN) [29], and multiclass logistic regression (LR) [30]. A practical model is then constructed using the most common data measured in each session based on the maximum score per session per patient. Unsupervised learning methods are then applied to the training dataset, such as the correlation matrix and principal component analysis (PCA), to show that all variables collected are relevant to the study. After performing dimensionality reduction analysis, the models are selected using 10-fold cross-validation on the training dataset with the mean and SD of accuracy from each computational experiment. Afterward, the following metrics determine the model’s performance, including the accuracy, precision, and recall from the confusion matrix. The macroaverage *F*_1_-score was used to judge the efficacy of the models, as this is a multiclassification problem [31], and as such, accuracy would be an insufficient measure. Figure 2 provides a high-level overview of the data collection, analysis, processing, and modeling that ultimately produces the final classification results.

### Details of Data Collection

Throughout a therapy session using the Motus Hand or Motus Foot, live angle data (measured in degrees from a natural midpoint in wrist or ankle placement) are collected from the sensor embedded in the wrist or ankle joint at 30 readings per second. These “raw” angle sensor data are then stored in a time series database (InfluxDB [32]). In addition to the high-resolution angle data, pressure readings (measured in PSI) are taken from the pressure management system at 30 measurements per second. While these readings are not high resolution compared to state-of-the-art kinematics technology [33], it is significantly higher resolution than what a typical physician would have access to during assessments in a normal physical therapy visit.

Each therapy session for a patient includes a selection of about 30 activities that focus on several types of motor function, including gross motor control, fine motor control, flexor tone reduction, endurance, reaction time, and tracking. A patient can participate in more than 1 video game (therapeutic activity) during a patient session. The score is recorded and stored once the patient completes the video game. The scores for each game are not necessarily standardized. This means a score of 100 in one game can represent a dramatically different performance than a score of 100 in another. The score is collected each time a player performs an action in the game that would increase or decrease the score, so this field is collected more irregularly and infrequently than angle and pressure data. Gender and other biometric data such as age, height, and weight are not included in the patient description or the analysis.

### Clinician Labeling

To train a classifier for determining stroke residual severity, our dataset must have appropriate labels corresponding to the patient’s level of function around the time the data were collected. During a series of video calls and using the Motus Hand and Motus Foot technology, a clinician met with each individual and performed a series of assessments. Remote assessment of extremity function using an external device has been studied and indicates that it is noninferior to in-person assessment when done properly [34]. The clinician used the potentiometer [35] embedded in the wrist or ankle joint of the Motus Hand or Motus Foot and a “clinician dashboard” interface to read live angle and pressure data from the patient and provide them the requisite assistance to stretch the patient’s wrist or ankle to collect passive and active ROM thresholds. With these assessments, the clinician estimated each individual’s active ROM and passive ROM and characterized their level of function as “no ROM,” “low ROM,” or “high ROM.” While these labels are quite broad, the labeling process is hardly a simple algorithm. At the clinician’s discretion, quantitative and qualitative factors must apply an appropriate label. In particular, the clinician recorded the minimum or maximum angle reached for the assessment performed, the type of assessment performed, and a label summarizing the patient’s ROM level. These data are summarized in Table 1.

**Table 1:** Example patient label table assessed by a clinician during telehealth session.

Patient ID	Maximum angle	Minimum angle	Assessment	Clinician classification
1495	37	–20	Passive	No
2273	21	–16	Passive	No
2085	40	–15	Passive	No
2098	44	–9	Passive	No
1864	28	–12	Passive	No
2040	37	–18	Passive	No
2097	43	–18	Passive	Low
2356	–3	–17	Assisted	Low
2356	–3	–17	Assisted	Low
1688	52	–23	Assisted	Low
1876	54	–12	Passive	Low
2029	46	–20	Passive	Low
1458	30	–18	Passive	Low
1637	10	–12	Assisted	High
2282	8	–16	Assisted	High
1781	39	–15	Assisted	High
2360	10	–18	Assisted	High

All patients first were given a passive ROM assessment, in which they were stretched as far as their wrist or ankle would allow without experiencing pain or discomfort. Next, an active ROM assessment was conducted. In this assessment, the patient bends their wrist or ankle as far up and down as they can without any assistance from the Motus Hand or Motus Foot and without compensating with other parts of their bodies (hips, shoulders, etc). Depending on the patient’s assessed active ROM, an assisted ROM assessment was performed. This assessment consists of providing patients with varying amounts of assistance and recording their ROM in the presence of an upward force.

We define a patient as “assisted” or “passive” based on the most arduous assessment performed on the patient. The low ROM label contains a combination of patients who either did or did not have enough movement for the assisted ROM assessment. All patients who are classified with a high ROM (low residual stroke severity) were able to complete the assisted ROM assessment. This is important when noticing that patients with ID 2085 and 1781 (blue) have a similar total ROM (maximum angle–minimum angle), but patient ID 1781 requires clinician assistance to reach their maximum ROM. However, there is ambiguity in some labels. For example, take patient ID 2356 (red), where it can be argued that the patient should have a high stroke residual severity (corresponding to low or no ROM), given the low total ROM with assistance. This is where the clinician has other outside factors that contribute to the final labeled classification of a patient. The clinician is visually able to assess the level of tone and spasticity that a patient may be exhibiting, which would not necessarily be captured in the minimum and maximum ROM values. The assessment results and labels were reviewed and confirmed by an additional expert.

### Data Processing

To create a more manageable dataset for the labeling task, we generate summary statistics of the high-resolution data for each activity performed during a therapy session. First, to compensate for sensor reading issues, we smooth outliers out of the raw time series data (replacing data points in the 99th and 1st percentile with the value of the 99th and 1st percentile, respectively). Then, summarize the angle (relative to a reference midpoint in degrees) and pressure (in PSI) using the following variables: *R_min_*, the minimum ROM for a game; *R_max_*, the maximum ROM for a game; *R_mean_*, the mean ROM for a game; *P_min_*, the minimum pressure for a game; *F_flex_*, the maximum centripetal force generated while moving downward (flexion for upper extremities and plantar flexion for lower extremities); *F_ext_*, the maximum centripetal force generated while moving upward (extension for upper extremities and dorsiflexion for lower extremities); *P_max_*, the maximum pressure for a game; and *P_mean_*, the mean pressure for a game. We finally pair these game-level summary statistics with the number of movements performed in the game (*N_mov_*), the maximum score in the game (*Score*), and the total time spent playing that game during a therapy session (*t_game_*).

This transformation from high-resolution data to game-level summary statistics provides a much more manageable dataset to which we can apply the clinician labels. A patient with low ROM (as labeled by the clinician) has little ROM during each game throughout a session. Using this idea, we construct a new dataset from each activity (game) a patient takes part in during a session, with each row having a unique (patient ID, session ID, and game ID) tuple. It is worth noting that a patient is unlikely to take part in every activity over the course of a therapy session; often, they gravitated to a few choice activities during each session. A summary of the data in each row in the described dataset is presented in Table 2.

After combining the data into this standardized dataset, the data then require sanitization, analysis, and normalization. The “role” column indicates whether a variable is part of the feature set, the labels, or not used in the model training at all. To sanitize the data, we fill in missing values, correct invalid sensor values, and throw out data that did not represent a meaningful therapeutic exercise.

To isolate games with insufficient activity to draw meaningful conclusions, we restrict the number of movements, *N_mov_*, performed during a game (therapeutic activity). A “movement” is any change of direction recorded in the angle sensor after noise is smoothed out of the time series. We remove any game (therapeutic activity) with fewer than 3 movements, as no significant therapeutic exercise can be performed with fewer than 3 movements (under assistance from the robotic Motus Hand or Foot).

Before performing any data analysis, the harmonized dataset is partitioned using an 80%:20% split into a training set and a holdout test set. The training set is used for exploratory data analysis and model training. All normalization and transformation techniques derived from the training set are then applied to the test set before the final predictive measures are computed. This is done to prevent data leakage from including the test set in the derivation of normalization and transformation techniques. The test dataset is reserved for the final performance measures in the Results section.

**Table 2:** Session game data dictionary.

Variable	Description	Role	Unit	Example
*F*_*flex*_	Maximum centripetal force generated moving in the downward direction during an activity (computed from derivatives of angle data)	Feature	Newton	–3.047709105
*F*_*ext*_	Maximum centripetal force generated moving in the upward direction during an activity (computed from derivatives of angle data)	Feature	Newton	3.251405759
*N*_*mov*_	The number of completed movements during an activity	Feature	Integer	10
*R*_*min*_	Absolute minimum angle detected by angle sensor during an activity	Feature	Degrees	–25
*R*_*max*_	Absolute maximum angle detected during an activity	Feature	Degrees	46.41941
*t*_*game*_	Total time spent performing therapy during an activity	Feature	Seconds	15
*P*_*min*_	Minimum pressure applied by the sensor during an activity	Feature	PSI	–0.04511994
*P*_*max*_	Maximum pressure applied by the sensor during an activity	Feature	PSI	10.30989
*P*_*mean*_	Average pressure applied by the sensor during an activity	Feature	PSI	3.590553432
*Score*	Maximum score achieved during an activity	Feature	Integer	100
*h*	Peripheral type variable indicating the hand or foot	Feature	0, 1	Hand
*Class*	Designate stroke severity label by a clinician (high, low, and no)	Label	0, 1, 2	High
*g*	Unique identifier for each game (therapeutic activity) that is available on the Motus Hand or Foot	Not used	Integer	4
*p*	Anonymous identifier for each patient using the Motus Hand or Foot in this study	Not used	Integer	11
*s*	Unique identifier for each session performed on the Motus Hand or Foot	Not used	Integer	782,302,348,734

### Exploratory Data Analysis

#### Data Distribution

**Figure 3:**
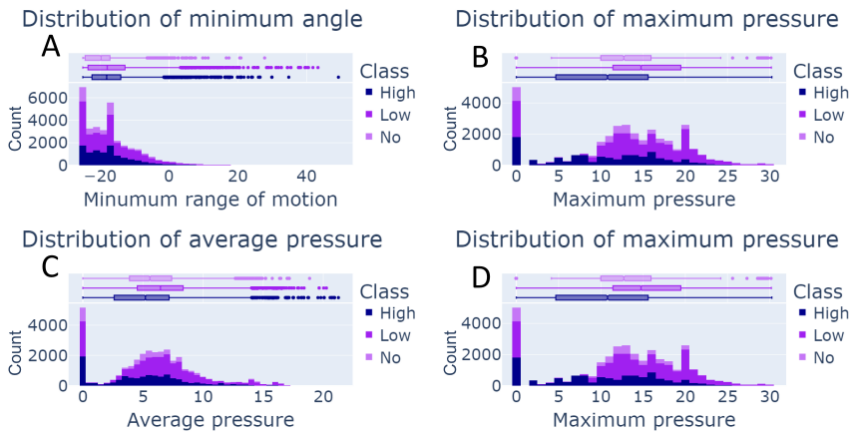
Example of the distribution plots for 4 of the random variables for each therapeutic activity color-coded by the class label from the clinician. The plots are (A) distribution of minimum angle, (B) distribution of maximum pressure, (C) distribution of average pressure, and (D) distribution of maximum pressure. An explanation for each variable in the dataset is given in Table 2. Subsequently, random variables are distributed normally, which is crucial for using the z score when inputting into a machine learning algorithm.

It is well-known that proper data normalization is critical for maximizing model performance across machine learning applications and methods [36]. Knowing the proper normalization technique for each feature requires a cursory dataset analysis. In Figure 3, we show representative distributions of the features that will be input variables for our comparative model analysis. While some variables are not normally distributed, assuming that the data are normally distributed is sufficient considering the results [37].

#### Correlation

**Figure 4:**
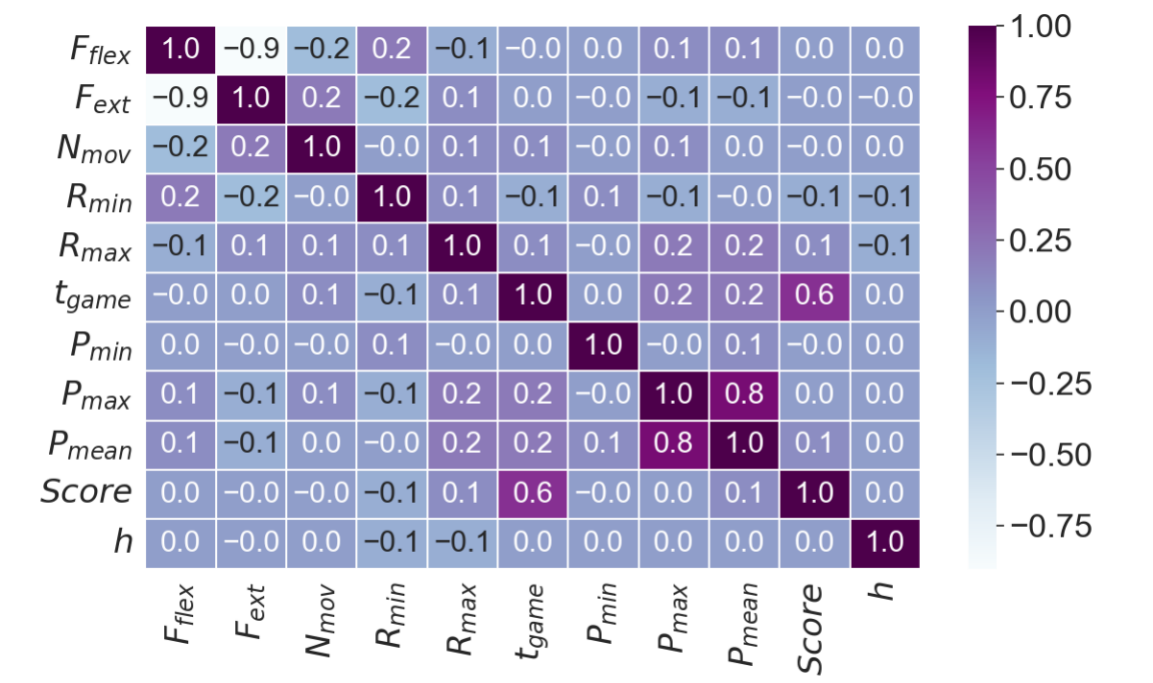
Correlation matrix for the feature set, excluding identification variables such as patient ID, session ID, game ID, and start time. A correlation threshold of |*Cor(X, Y)*|>0.9 was used for variable exclusion in dimensionality reduction. Notably, *F_flex_* and *F_ext_* show a strong negative correlation. Despite this, both were included in the analysis because the development of downward pushing strength (flexion in upper extremities and plantar flexion in lower extremities) does not imply the development of upward pushing strength (extension in upper extremities and dorsiflexion in lower extremities).

We analyze the correlation among the features in our dataset to identify potential redundancies. Then, we look at the principal component decomposition [38] to see if the variation in the data can be meaningfully reduced to a lower dimensional space. The correlation matrix for the feature set, constructed by computing the correlation between each pair of features in the dataset, is shown in Figure 4.

Because a correlation matrix points to potential relationships between features, it can indicate the feasibility of dimensionality reduction when preparing a dataset for building a classifier. If 2 variables are highly correlated, that is, |*Cor*(*X, Y*)|*>*0*.*9, Shin and Park [39] suggest that one of those variables can be dropped from the analysis. We use this threshold of 0.9 where appropriate.

There exists a strong negative correlation between F_ext_ and F_flex_ approximately at –0.9. However, we chose not to exclude either variable from our analysis due to their relevance in neuromotor recovery. For survivors who have experienced a stroke with upper extremity impairment, hypertonia often results in distinct patterns of volitional flexion (downward pushing force) and extension (upward pushing force) improvement [40].

The correlation between the mean pressure for an activity, *P_mean_*, and maximum pressure for an activity, *P_max_*, with the value of 0*.*80, indicates that the Motus Hand or Foot applied more pressure on average in each activity; however, because this correlation fails to surpass the threshold of 0.90, we do not drop either variable. Similarly, the correlation (0*.*60) between game time, *t_game_*, and game score, *Score*, is intuitive: the longer a patient plays a game, the higher their score. Unfortunately, this correlation also does not meet the threshold for exclusion in the final feature set.

#### Dimensionality Reduction

Another informative approach for analyzing the potential for dimensionality reduction in a feature set is PCA. Principal components are new variables constructed as linear combinations of the initial variables. These linear combinations ensure that the new variables (ie, principal components) are uncorrelated and that as few components as possible contain most of the information from the initial variables. Explained variance is a statistical measure of how much variation in a dataset is attributable to each principal component (eigenvectors) generated by the PCA method [41]. Explained variance thus allows us to rank the components in order of importance and to focus on the most important ones when interpreting the results of our analysis.

**Figure 5:**
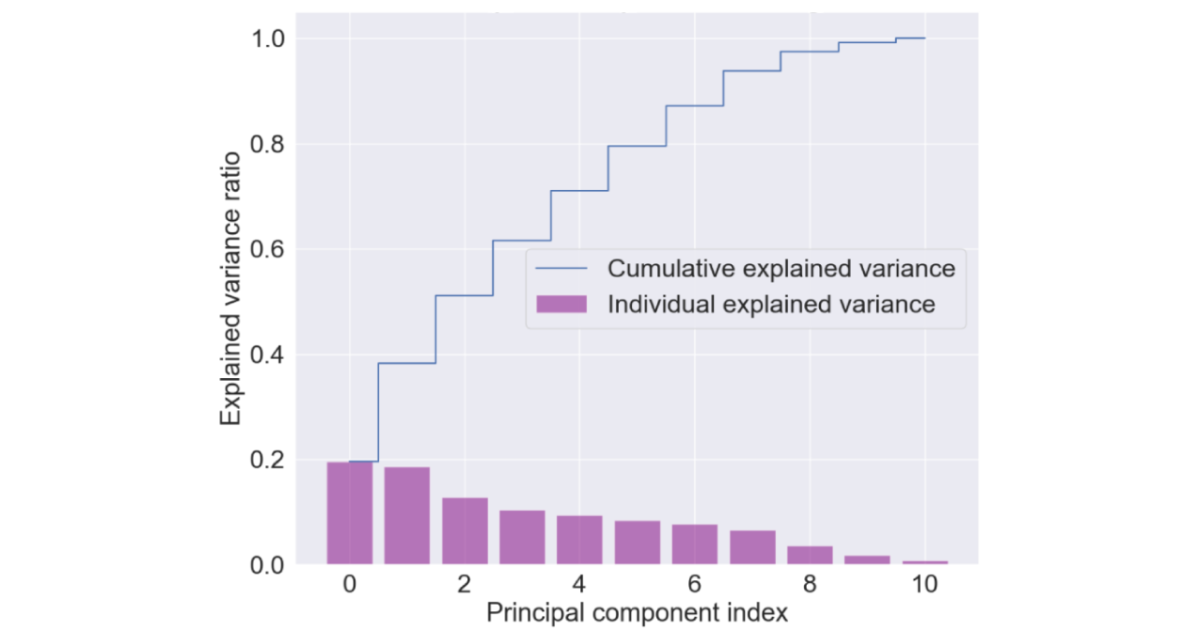
Depiction of the principal components with the explained variance ratio. As shown, 95% of the explained variance is contributed by all principal components. As a result, all variables are used in the machine learning model for the analysis.

In Figure 5, we show the explained variance of each principal component contributes to the total variation in the feature set. No component can be described as dominant, as none accounts for more than 20% of the variance in the initial dataset.

Given this and the results from our correlation analysis, no variables present in the principal dataset were excluded from the feature set.

### Model Description

Here, we provide a brief overview of the models compared in the *Results* section. LR is a classical statistical technique for binary classification. The technique consists of mapping the probability of an event happening to a logistic curve with the model inputs as dependent variables. LR is still widely used and is a common first model when performing classification because it is easy to implement and interpret.

Gradient boosting decision tree (GBDT) is a widely used machine learning algorithm due to its efficiency, accuracy, and interpretability [27]. The algorithm uses smaller “weaker classifiers” with a number of leaves. By taking a weighted average of these several “weaker classifiers,” we then can construct a “stronger classifier” [42]. By training several weaker models, this process is known as AdaBoosting. It results in a stronger model by adding more leaves to the decision tree and taking a weighted combination of these weaker models, where the weights are determined by the performance [43].

The DNN is a high-performance deep learning model with varying hidden layers. Several architectures were tested on the training dataset to see if there was an increase in performance by adding hidden layers (from 4 to 8) or a reduction in nodes in each input layer [44]. The rectified linear unit activation function was implemented into the model instead of the sigmoid function. Both were tried. Accuracy results from the computational experiment could surpass 80%, regardless of adding more layers, changing the hidden layer input size, or changing the activation function. The best-performing DNN trained in our analysis has 3 hidden layers with the input size of the hidden layers as 8, 5, and 8, respectively. Layer size, learning rates, batch size, and epoch size were all hyperparameters tuned during the training process.

The extra trees classifier is an ensemble learning method for classification. Ensemble learning is a machine learning technique that combines the predictions of multiple individual models to produce a more accurate and robust final prediction. The basic idea is to train multiple models independently, each with a different algorithm or set of hyperparameters, and then combine their predictions at the end [45]. This is similar to the AdaBoosting concept with LGB, where models can be combined by averaging or weighting their predictions [46]. The model uses entropy as the splitting criterion for the trees, with 100% of the features considered at each split. The maximum number of leaf nodes for each tree is 8717, and the model is comprised of 42 trees [46].

### Ethical Considerations

This study was approved by the institutional review board of Georgia State University (IRB H24270). This research involves the analysis of preexisting, nonidentifiable data. No direct interaction or intervention with human participants occurred during the course of this study. The study relies solely on data generated by the commercial company Motus Nova for nonresearch purposes. All methods followed relevant guidelines and regulations approved by the Georgia State University institutional review board that waived the informed consent and designated this study as no human participants research.

## Results

The original harmonized dataset (described in Table 2) contained all the scores, the minimum and maximum ROM, and minimum and maximum pressure, and we took the maximum score per game per session. Due to the smaller dataset, the training and testing were split on the 80%:20% principle, where for 80% of the data, the training set was used to train the models (with a subset of this set being used for training hyperparameters where appropriate). For the remaining 20%, the holdout test set was used to compare the performance of the models after training.

Table 3 shows a performance based on 10-fold cross-validation for each machine learning classification algorithm applied to the training set. K-fold cross-validation is used to verify that a high-accuracy model does not necessarily overfit the training data. The training dataset is randomly divided into 10 different subsets or “folds” [47]. Each of these folds is then used as the new training data, while another is used as the new testing data for fitting a new model. We then take the mean and SD of the model accuracy across the 10 folds.

From Table 3, it is clear that both the neural network and LR perform poorly compared to the tree-based methods (extra trees classifier and LGB). The poor performance for LR is likely due to the assumption that there is a linear relationship between the features and the labels, that is, the points corresponding to each label can be nicely separated by a hyperplane (the N-dimensional extension of a line in 2D or plane in 3D). On the other hand, neural networks tend to perform poorly on small datasets like the therapy dataset we have compiled. This is because, while able to capture nonlinear decision boundaries, neural networks are prone to overfitting the training dataset. Tree-based methods provide an excellent combination of low bias but are still able to capture a nonlinear decision boundary.

**Figure 6:**
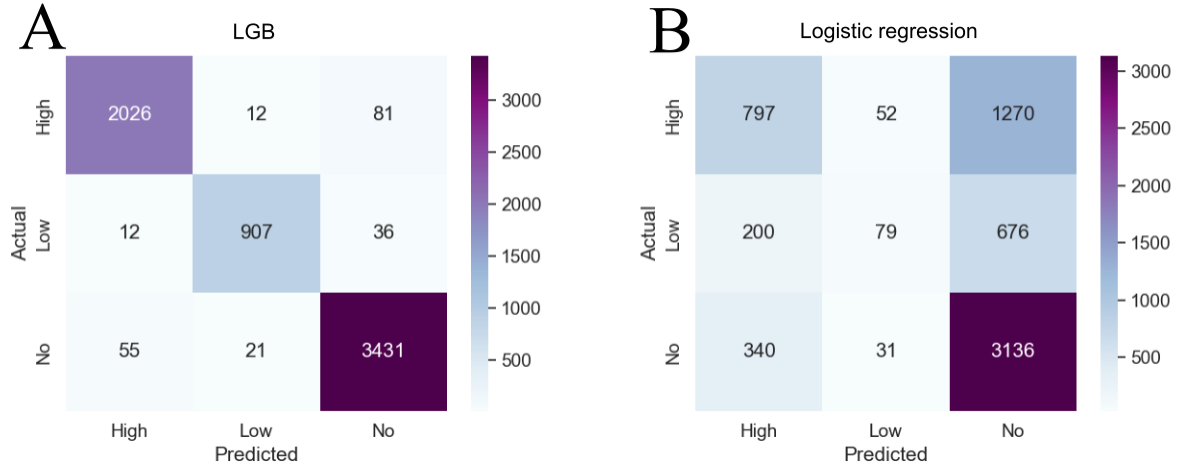
Confusion matrices for (A) light gradient boosting (LGB) and (B) logistic regression. Considering the false negative column of the no classification, it is seen that the LGB model greatly improved this classification. This is especially important when classifying a patient as having “no” stroke severity when they are actually a “high” severity. Misclassifications can be particularly dangerous, ranging from providing inadequate therapy to a patient with high ROM to injuring a user with low ROM with therapy designed for a user with high ROM. ROM: range of motion.

Figure 6 presents the confusion matrix of each of the supervised learning methods. A confusion matrix is used to represent the algorithm’s performance visually. Each row of the matrix represents the instances in an actual class, while each column represents the instances in a predicted class or vice versa. We represent the percentage over the exact numeric number for display purposes. Three performance metrics come from the confusion matrix: precision, recall, and the *F*_1_-score. Accuracy measures the proportion of predicted positives that are truly positive. Recall measures the proportion of predicted negatives that are truly negative. To compare the performance of each model, we use the *F*_1_-score. The *F*_1_-score is the harmonic mean of the precision and recall [31]. In this case, this is macroaveraging (treating all classes equally important).

A full breakdown of the performance measures (precision, recall, and *F*_1_-score) for all models on the holdout test set is shown in Table 4. It is important to notice that while the extra trees classifier has a comparable accuracy (picking the correct label) with the LGB method, LGB performs reliably better than all of the other models when also weighing false positives and false negatives (precision, recall, and *F*_1_-score). Remarkably, the LGB model best fits the dataset with a weighted average *F*_1_-score of 96.70% compared to LR (55.82%), extra trees classifier (94.81%), and DNN (70.11%).

**Table 3:** Training set cross-validation algorithm accuracy.

Algorithm	Accuracy (%), mean (SD)
Extra trees classifier	96.40 (0.4)
Light gradient boosting	94 (0.4)
Neural network	71.70 (0.7)
Logistic regression	61.20 (0.5)

**Table 4:** Performance measures on the holdout test dataset for each model for each label type.

Model and label			Performance metric				
			Precision (%)		Recall (%)		*F*_1_-score (%)
**Extra trees classifier**							
	Low	95.44		92.83		94.12	
	High	94.46		91.10		92.75	
	No	94.55		97.04		95.78	
	Weighted average	94.82		94.84		94.81	
**Light gradient boosting**							
	Low	96.80		95.61		96.20	
	High	96.49		94.97		95.73	
	No	96.70		97.83		97.26	
	Weighted average	96.70		96.70		96.70	
**Deep feed-forward neural network**							
	Low	74.34		64.94		69.32	
	High	61.93		28.27		38.82	
	No	71.64		87.71		78.86	
	Weighted average	71.14		71.94		70.11	
**Logistic regression**							
	Low	59.61		37.61		46.12	
	High	48.77		8.27		14.15	
	No	61.71		89.42		73.02	
	Weighted average	59.15		60.96		55.82	

## Discussion

### Principal Findings

We have demonstrated that objectively measured rehabilitation training combined with machine learning methods can be effectively used to identify residual stroke severity classes. This approach aims to enhance in-home, self-guided, individualized stroke rehabilitation. We have tackled several challenges commonly faced in health care applications of machine learning, such as processing data with varying physical quantities, handling errors in sensory data, and addressing ambiguous classifications due to human error.

### Comparison to Prior Work

Previous studies have largely focused on predicting short- and long-term functional ability based on clinical variables from an inpatient hospital stay immediately after stroke [16-18] or they have used robotic measurements to predict clinical measurement scores [48,49]. Meanwhile, our study focuses on data collected during rehabilitation in the in-home setting that is used to predict residual stroke severity.

In those previous studies, the algorithms most frequently used were linear and LR. However, these methods showed poor accuracy (less than 80%) with our dataset, prompting us to explore different approaches. We found that the LGB method provided substantially higher accuracy, despite being applied to a relatively small dataset. LGB is advantageous for real-time autonomous stroke residual severity classification; it is known to be easily transferable and requires relatively little computational resources [27].

### Strengths and Limitations

Our study design ensures that the model can make decisions based on summary statistics typically available in an outpatient rehabilitation setting. This design enables retraining of the model in an outpatient environment, similar to an in-home setting. Consequently, the model can offer a second opinion on a patient’s stroke residual severity or potentially replace a clinician’s assessment. This capability allows for a more targeted therapy routine based on the stroke residual severity classification.

A notable limitation is that the model may need retraining to accommodate specific data collected in each outpatient setting, accounting for differences in the data. Such data collection can be challenging and costly, particularly for outpatient facilities with low patient volumes.

### Future Directions

Future work involves building an expanded and more sophisticated dataset. Real-time processing of sensor data will enable a classifier to interact with users in real time, recognizing and classifying subtle changes in their motor function. This capability will allow clinicians (AI or otherwise) to prescribe personalized, targeted interventions that are most impactful.

Additionally, integrating real-time understanding of a patient’s needs with an in-home robotic therapy device like the Motus Hand and Motus Foot will provide immediate feedback. An AI in therapeutic games can detect patient needs, such as fatigue, during a therapy session and adapt its strategy accordingly. Further research could also explore finer-grained severity classifications, such as labeling patients based on their total ROM or amount of tone, which would require more labeled data to train the machine learning model properly.

### Conclusions

Autonomous classification is becoming more important for successful rehabilitation, as rehabilitation begins to move out of the clinical setting. Still, it faces challenges with the accessibility and volume of appropriate clinical data for training models and model access to user data for classification.

By leveraging the in-home stroke rehabilitation robotics provided by the Motus Hand and Motus Foot, we have made significant progress in addressing these issues that prevent adequate training of an autonomous classification model. With the data collected from self-guided, in-home therapy sessions, we could train a classification model to identify the stroke residual severity in 33 patients. We compared 4 different models: extra trees classifier, LGB, DNN, and LR, finding the LGB method to outscore the other 3 with an average *F*_1_-score of 94%. The LGB method is a particularly powerful model for this case because it combines interpretability and portability.

Because our model relies only on therapy session summary statistics, the proposed method is expected to be successful when applied to comparable rehabilitation datasets. Once trained, the model is highly portable and can be integrated into similar rehabilitation settings, such as outpatient rehabilitation facilities with appropriate technological resources, to provide an autonomous real-time classification of stroke residual severity. Additionally, when paired with something like the Motus Hand and Motus Foot technology, our classifier provides the opportunity to develop personalized training based on the stroke residual severity of the individual and adapt the therapy exercises to each patient’s needs. The efficacy of real-time classification and adaptation remains a subject of future study.

## Abbreviations

AI: artificial intelligence
DNN: deep feed-forward neural network
LGB: light gradient boosting
LR: logistic regression
PCA: principal component analysis
ROM: range of motion

## Data Availability

The datasets generated and analyzed during this study are not publicly available because they are owned by Motus Nova, LLC, but are available from the corresponding author on reasonable request.
